# Model for Predicting the Effect of Sibutramine Therapy in Obesity

**DOI:** 10.3390/jpm14080811

**Published:** 2024-07-31

**Authors:** Sergey D. Danilov, Georgiy A. Matveev, Alina Yu. Babenko, Evgeny V. Shlyakhto

**Affiliations:** Laboratory of Prediabetes and Metabolic Disorders, WCRC “Centre for Personalized Medicine”, Almazov National Medical Research Centre, Saint Petersburg 197341, Russia; sergey.danilov@niuitmo.ru (S.D.D.); alina_babenko@mail.ru (A.Y.B.);

**Keywords:** obesity, prediction model, sibutramine, XGBoost classification, Shapley data valuation

## Abstract

**Background:** The development of models predicting response to weight loss therapy using sibutramine is found in only a few cases. The objective of the work is to develop a data-driven method of personalized recommendation for obesity treatment that would predict the response to sibutramine based on the current set of patient parameters. **Methods:** The decision system is built on the XGBoost classification algorithm along with recursive feature selection and Shapley data valuation. Using the results of clinical trials, it was trained to estimate the probability of overcoming a weight loss threshold. The model was evaluated by the accuracy metric using the Leave-One-Out cross-validation. **Results:** The model for predicting response to sibutramine treatment over 3 months has an accuracy of 71%. The model for predicting outcomes at the sixth month visit based on results at 3 months has an accuracy of 80%. **Conclusions:** Although our developed prediction model may not exhibit high precision compared to certain benchmarks, it significantly outperforms random chance or models relying only on BMI parameters. Our model used the available range of laboratory tests, which makes it possible to use this model for routine clinical use and help doctors decide whether to prescribe sibutramine.

## 1. Introduction

Obesity is one of the most common non-communicable pandemics and, with its visceral nature, it is naturally accompanied by insulin resistance and, subsequently, the development of diabetes mellitus (DM), arterial hypertension (AH), and dyslipidemia (DLP) (hypertriglyceridemia and a decrease in high-density lipoprotein (HDL) concentration). Since obesity is the cornerstone of this cluster of metabolic pathologies, weight loss is a key task in their correction. Thus, a 5% weight loss can make a huge contribution to improving metabolic health and become an impetus for further active weight loss [1]. This parameter (5% weight loss) is included in the obesity treatment algorithms as the minimum value indicating the effectiveness of treatment [2]. Unfortunately, none of the existing methods provide effective weight loss in 100% of patients, which requires a personalized predictor-based approach to choose the most effective treatment method for each patient.

To solve this problem, artificial intelligence (AI) with machine learning models (MLMs) has been actively studied and applied in recent years. MLMs make it possible to predict the response to therapy, and their implementation in healthcare can significantly increase its effectiveness [3,4]. Based on the data from literature reviews, it was shown that the majority of studies (in 18 of 22 studies) using AI-based models showed higher prediction accuracy compared to traditional statistical approaches [5]. Another important task that MLMs can solve is minimizing the time constraints related to a therapy choice. The time for making a decision is limited during an outpatient visit to a doctor. For the optimal choice of individualized therapy for each patient, it is necessary to consider a multitude of factors. During the outpatient appointment, the specialist should have time to interview the patient, enter the patient’s information into the medical information system (MIS), schedule the patient with other specialists if necessary, and write referrals for examinations and prescriptions for medications, having previously assessed the presence or absence of contraindications to a particular class of drugs and weighing the chances of successful treatment. In this situation, computer-based decision support systems (DSSs) based on AI and MLMs can significantly reduce time costs and optimize choices. At the moment, MLMs are used in various fields of medicine. In radiology and oncology, artificial intelligence can help characterize the findings on images, automatically identify and classify them as benign or malignant, and calculate and plan radiation therapy [6]. In cardiology, machine learning significantly increases the accuracy of predicting cardiovascular risk, increasing the number of identified patients who may benefit from preventive treatment [7]. In endocrinology, DSSs are used to assess the risk of developing diseases, particularly diabetes, in individuals to ensure timely and optimal preventive measures [8]. DSSs are also used to evaluate the achievement of target values for primary metabolic parameters (they can be intended for both the doctor and the patient), for which data from activity tracking bracelets, electronic nutrition diaries, and devices monitoring glycemia are used. For example, the DiaCompanion application developed by our colleagues for patients with gestational diabetes [9] predicts changes in blood glucose concentration after taking a particular meal, allowing patients to adjust their food plans and prevent hyperglycemia. DSSs can also help in selecting the optimal type and volume of therapy during outpatient visits [10]. Finally, MLMs and DSSs based on them can be useful in predicting the risk of complications, death, and choosing the optimal volume and time of correction in urgent situations for both in patients with diabetes and in the general population. As an example, Oliveira et al.’s research can be indicated, as well as that of Hadanny et al. [11,12]. There is evidence showing the effectiveness of using artificial intelligence in decision support for various dietary interventions in obesity management [13,14,15,16]. The use of machine learning techniques to study the interrelationships between psychological components (anxiety, depression) and changes in BMI was evaluated, and a high degree of predictive precision of the result was shown in one previous study [17]. There is evidence that in-depth phenotyping of obesity can potentially determine therapeutic planning for various subtypes of obesity [18]. However, the use of AI to predict the response to drug therapy for weight loss is just beginning and the data in the literature are limited in scope and not presented for all drugs. In particular, the development of such models using sibutramine is described in only two studies [19,20].

Previously, Derevitskii and a team of medical experts from Almazov National Medical Research Center proposed a system which allows medical specialists to calculate the future effects of the weight loss drug sibutramine [19]. The authors implemented a binary classification model with a successful prediction accuracy of about 86%. On the other hand, the author of the second model uses hard-to-access and expensive analyses such as GLP-1, ghrelin, and others. This makes prediction on the first visit very difficult, which does not suit our goal of making weight loss forecasts based on a limited number of parameters. Continuing this research, the utilization of regression modeling was suggested to predict specific outcomes in a subsequent study [20]. Furthermore, the methodology was extended to several medications: sibutramine, metformin, and liraglutide. However, the set of patient parameters used in the models does not also meet the target requirements for the research. In the case of a shortened, minimally necessary, and feasible set of parameters for collection, no additional methodologies are provided to achieve sufficient quality. No research on performance decline is provided for a situation where we use a smaller subset of parameters when a full set of parameters is not available. Consequently, as a main aim in developing the methodology, we can declare the ability of a model to provide good accuracy with small, noisy, and limited-in-predictor-variables data.

For tasks involving small datasets, complex models may not always be the best choice due to the risk of overfitting. In such scenarios, tree-based models, such as decision trees, random forests, and gradient boosting machines, have consistently demonstrated superior performance. These models excel at capturing complex relationships within the data while remaining interpretable and robust. Despite the growing popularity of deep learning methods, tree-based models continue to outperform them on typical tabular data [21], especially when datasets are small. Moving from model architecture improvement, we can investigate solutions related to the valuation of training data and filtering. This includes the identification of label errors, and filtration of noisy, harmful, or uninformative data with a negative impact on model quality. The Data Shapley method [22] sufficiently improved the Alzheimer’s disease factors prediction on MRI scans according to the recent research [23]. This method is commonly used for filtering wrongly labeled data, noisy samples, or mistakes. The applicability of this approach has been proven on the dataset with 756 images, and applicability for small data, as in this project, has not been previously researched, although it may seem that in small data no samples can be removed. Therefore, we can experiment with the methodology and integrate it into our task to analyze the ability to improve the accuracy of weight loss prognosis for patients. There are some improvements of this method [23,24], but according to guidelines and experiments from the study, the classical Data Shapley method perfectly fits the data valuation and filtration tasks, especially when researchers work with small datasets.

The system we are developing is designed to serve two purposes. On the one hand, it is intended for diabetes prevention, as it is well established that weight loss is crucial for achieving this goal. On the other hand, it enables the optimization of the selection of medication therapy for obesity, specifically the appropriateness of prescribing sibutramine. The system is expected to predict future success within three months of treatment, and, after this period, suggest to the doctor whether the patient should continue the chosen strategy or not. According to limitations and current trends of working with small data, the methodology should be focused on preprocessing the data and selecting the best clinical records to achieve high precision in the prediction of future weight loss.

## 2. Materials and Methods

For the study, a sample of very well-phenotyped patients was used, collected on the basis of the Research Laboratory of Diabetology of the Institute of Endocrinology of Almazov National Medical Research Centre, who evaluated not only standard anthropometric and metabolic parameters but also the concentration of hormones involved in the regulation of energy balance and appetite (leptin, ghrelin, incretins (GPP1 and GIP)), their postprandial dynamics, biomarkers of inflammation in the bloodstream (CRP, myeloperoxidase, paraoxonase −1), and a number of molecular genetic markers, the expression of which was altered in subcutaneous adipose tissue during obesity (leptin, ADIPOQ, HIF1a, CCL2, miR142, miR155, miR378). Our previous studies demonstrated the existence of a relationship between these indicators and the response to treatment in obesity [24], which allowed us to assume their usefulness in constructing a predictive model.

The study was performed in accordance with the standards of Good Clinical Practice and the principles of the Helsinki Declaration. The research protocol was approved by the local Ethics Committee of the Almazov National Medical Research Centre (extract from Protocol No. 022018-14d dated 12 February 2018). Before inclusion in the study, written informed consent was obtained from all participants.

The inclusion/non-inclusion criteria are presented in Table 1. The study group of patients received sibutramine therapy (REDUXIN) at a dose of 10 mg/day (1 capsule) in the morning 30 min before meals, for a period of 6 months. Nutrition standardization was performed for patients included in the study: moderate hypocaloric intake of 15 kcal/kg (carbohydrates 45–55%, proteins 15–20%, fats 20–35%, fiber 35 g/day) or based on the ideal body weight according to the Mifflin–St. Jeor formula with adjustments for physical activity levels.

To exclude patients with liver pathology, a blood test was performed for ALT, AST, total bilirubin, renal pathology—creatinine, glomerular filtration rate (GFR; Cockcroft–Gault calculation), and pathology of carbohydrate metabolism—fasting plasma glucose concentration. The exclusion of pathology from the cardiovascular system was performed anamnetically (the presence of a history of myocardial infarction, stroke, chronic heart failure III-IV FC (NYHA), hypertension with inappropriate blood pressure levels).

Having collected the dataset, we continue research by pre-processing this data. As it was mentioned, the main goal of our decision system is to predict the most possible outcome of obesity treatment. So, we set binary labels as targets for the model corresponding to the treatment success thresholds at 5% for the 3-month period and 7% for the 6-month period. Additionally, we appended the result of 3 months of treatment in the model for the second visit after completing the first part of therapy. One of the key features of the system, as stated before, is the utilization of the most available and low-cost parameters in the clinical practice set. So, the model building process is divided into two stages: the described parameters are applied to the models, and then, to make the decision more stable, the expert appends scientific parameters (hormones, mRNA, and other tests). 

The data processing part included several parts. To mitigate the influence of outliers on the model, parameter values that fall outside the interval bounded by Q3 + 1.5 IQR (third quartile) from above and by Q1–1.5 IQR from below were replaced with the mean value of the parameter. For experiments with classifiers, apart from gradient boosting, missing values were filled with the mean for continuous variables and the mode for discrete variables.

The next step involves feature engineering, which includes various methods. We identified the predictors that have the most significant impact on the target variable according to statistical tests. The first method is correlation analysis, where for continuous variables we assessed the correlation with the percentage of weight loss using both Pearson and Spearman correlations. Additionally, we examined the distributional differences between success groups using Student’s *t*-test, the Mann–Whitney U test, and Kolmogorov–Smirnov test. For discrete variables, the Chi-Square test was employed. The parameters exhibiting a significance level (*p*-value) below 0.05 in these tests were selected for further analysis.

The next step was the subsequent removal of such features that do not impact model efficiency. We used the Recursive Feature Elimination algorithm with Random Forest [25], iteratively assessing the ROC AUC classification metric with and without each selected feature and removing those that did not positively impact the metric’s value. By using this technique, we were able to more precisely select a set of features with higher predictive ability.

Having chosen a set of optimal predictors, we moved on to sampling, where we should evaluate training data points and eliminate those with a negative impact on the performance of the model. Rather than using a classical simple Leave-One-Out sample removal method, which is unstable and not effective enough for real-world tasks, we used an advanced method for the valuation of the dataset called Data Shapley [22]. This approach utilizes Shapley values from game theory, where each simple from the training dataset has a role of a player in a game, and we assess how each player impacts the model performance. According to the calculated Shapley values, we remove samples that are less than or equal to pre-defined threshold values, and after each step, we evaluate the metrics. We used a Random Forest classifier as a model for performance change evaluation, and as shown in a paper on the method [22], the results we obtained on a simple model could be transferred to a complex model without loss of quality.

Turning to machine learning model training, we experimented with various classifiers, including Logistic Regression, Support Vector Machine, Decision Tree, Random Forest, and XGBoost. During those experiments, we faced challenges related to the high instability of performance metrics. To address this limitation, we employed the stratified 10-fold cross-validation technique to maximize the utility of the available data and check the robustness of the solution.

**Figure 1:**
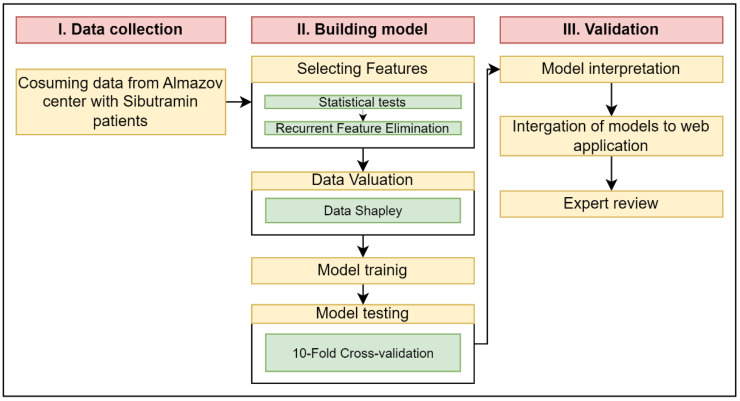
Pipeline of methods in model building.

After the model preparation steps, we studied the internal workings of the decision system by explaining the influence of individual predictors on the model’s solution. The necessity of explaining the model in this study is driven by several factors. According to good practices, ML models which are used in doctors’ decisions should be interpretable, and the doctor should know the details of the decision path where he or she can correct or add new data to make a forecast more confident. Additionally, the information about the local interpretation of the model’s work can lead us to formulate hypotheses about the effect of the drug on the process of weight loss. As a result, utilizing interpretation frameworks can make the decision process more clear, confident, and productive than blindly trusting an algorithm’s output. Therefore, in this research, we used the SHAP library [26], which is based on cooperative game theory, as in the data selection step, and assigns each feature an importance value, indicating how much and on which side (positive or negative) each feature magnitude outputs from the mean value to the given model’s prediction for that instance. These local explanations can provide experts with insights into the model’s work and meaningful patterns from data or processes. Finally, we combined all these methods into a pipeline (Figure 1) and performed experiments with data to select the best solution.

## 3. Results

In the feature selection, we chose parameters that correlate with the target variable, or in such a way where distribution of parameters is significantly different between success and failure cases. The results of statistical tests are presented in Table 2.

After that, we corrected the list of parameters by using the Recursive Elimination Algorithm and selected the optimal set of features, as described in Table 3.

**Figure 2:**
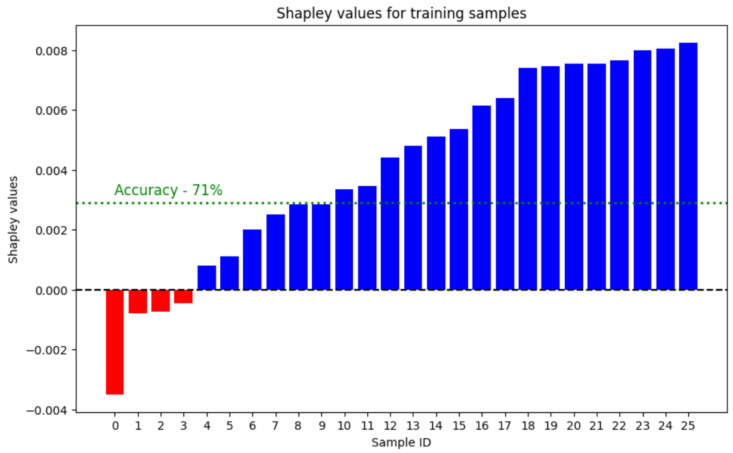
Result of the Data Shapley valuation of samples.

Sample filtering was performed only for the model with a 3-month forecast using a truncated set of parameters, as this configuration leads to the worst quality and requires advanced tuning. We evaluated the training samples using Data Shapley methods to identify noisy and unhelpful training data. Data valuation was made using the TMC–Shapley method [23] with Random Forest as a classification model in 4500 iterations, and the accuracy metric was used for validation on test split. The result is shown in Figure 2, where samples with the least Shapley values are presented. Accordingly, four samples from the training set have negative Shapley values and have a negative impact on model accuracy.

**Figure 3:**
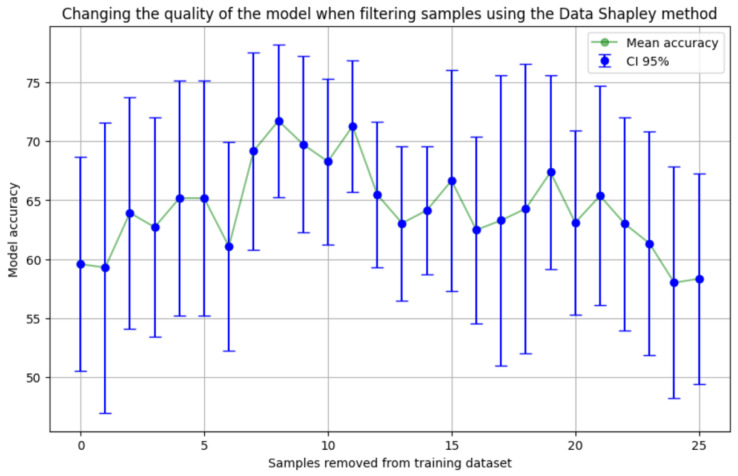
Accuracy after removing samples according to their Shapley value.

For a clear decision on the number of samples to remove, we iteratively removed samples in ascending order of Shapley values and evaluated the accuracy of the model trained with a new set of training examples. As shown in Figure 3, after removing eight samples, we were able to achieve improved accuracy up to 71.75% (±6.48%), and subsequent removal of samples did not further improve the quality.

As a result of the experiments, the best quality was shown by the classification model based on XGBoost gradient boosting. In addition to the quality indicator, the model was chosen for the following factors: it does not require additional preprocessing of values, including the insertion of missing values, and it is also adapted to local interpretation methods.

Accordingly, considering these facts and given accuracy, XGBoost was selected as a classification model for the application with the decision system and for further research on obesity treatment success factors. No additional hyperparameters tuning was performed, as the default configuration is well suited for the task. Table 4 presents corresponding metrics of the model (with 95% CI).

## 4. Discussion

Sibutramine was chosen as a weight loss medicine because our patients did not have significant cardiovascular pathology, and sibutramine has a tablet form of use and an inexpensive price, which is an important advantage over liraglutide.

In this study, models were created to predict the effect of sibutramine treatment with a prediction horizon of 3 months and 6 months. During model validation, it was determined that without using complex parameters such as GLP-1, GIP, and others, it is possible to obtain a model of sufficient quality for application in clinical practice. No other study can propose a model that works with a set of parameters and measurements that can be performed in a clinic setting within a maximum of two weeks with a limited budget. This comparative advantage can provide healthcare practitioners with greater confidence in treatment decisions, providing a valuable tool to improve patient care and outcomes.

In more detail, the model for 3 months without complex parameters had a classification quality of 71% for the XGBoost model in such a case, while high precision (76%) and recall (75%) metrics were observed for the “weight loss < 5%” class. From this result, it can be concluded that the model performs well in estimating the probability of unsuccessful treatment, highlighting factors that complicate weight loss. Conversely, the lower ability to accurately estimate positive treatment outcome is due to numerous factors, such as sport regime, and adherence to doctor’s prescription during obesity treatment, which could not be considered by the ML model.

**Figure 4:**
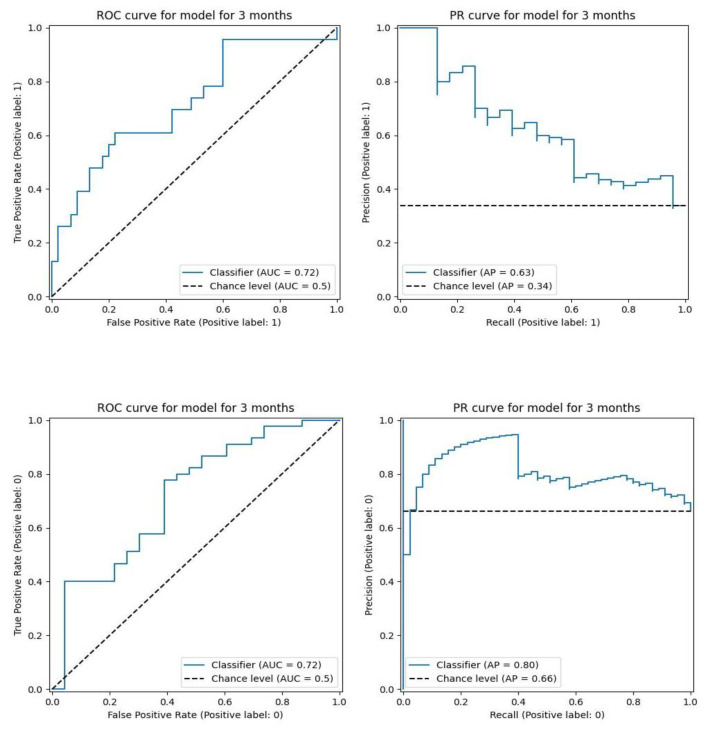
ROC curve and PR curve of model for 3 months without scientific parameters for labels 0 and 1.

Additionally, the models perform the classification task better than a random classifier, and they adequately utilize the input parameters. This can be demonstrated on the ROC and PR curve plots (Figure 4), and while comparing the performance of our model to the model using only the patient’s age and BMI, the quality is 12% higher.

For the convenience of medical professionals, the ability to predict further treatment progress based on 3-month weight loss results was added. For this type of model, 80% and 84% results were obtained with a different set of parameters. Using this prediction mode, the specialist will be able to adjust the treatment strategy and thus maintain the weight loss rate.

The addition of GLP-1, GIP, ghrelin, miR142, and sST2 parameters improved the quality of the classifiers, achieving 75% for 3 months and 84% for 6 months. Such models will be more useful for the work of a research scientist who seeks to understand the mechanisms of action of a particular drug at the hormonal and molecular genetic level.

**Figure 5:**
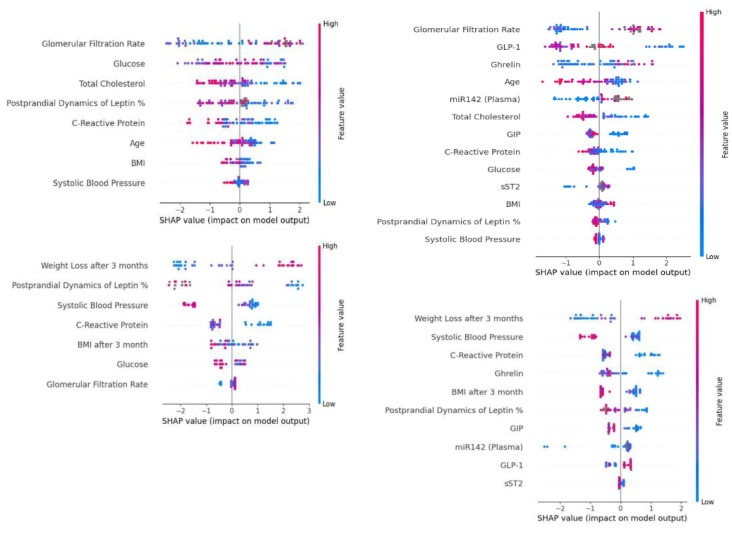
Interpretation of the models by the SHAP framework.

To make some hypotheses on the cognitive process of the model, we analyzed received explanation plots constructed with the SHAP framework, which uses Shapley values for evaluating the impact on the target result of the model. Results for all four models are shown on Figure 5.

**Figure 6:**
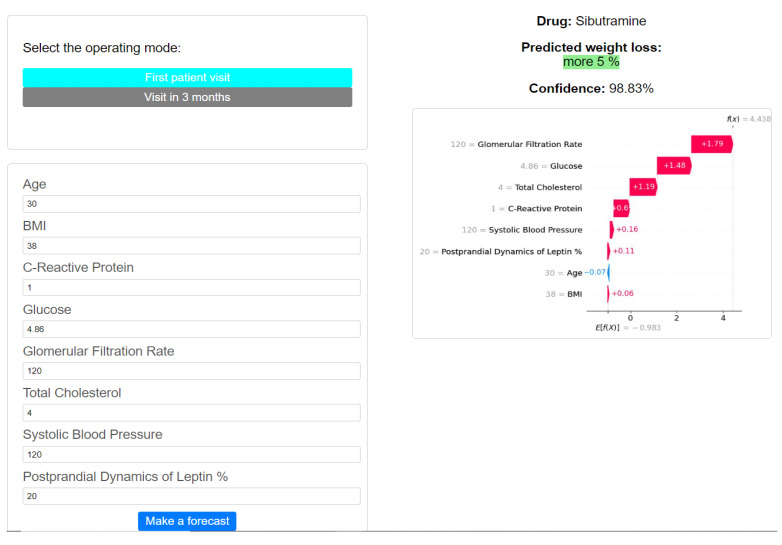
Example of usage of developed application for one of the real patients.

To utilize the trained ML models and interpretation algorithms, we developed a web application using Flask. This application loads pre-trained XGBoostClassifier models and, based on user input values, predicts treatment outcomes. Users can select between two modes: the first visit or the visit after 3 months, and they can specify whether parameters from the third group (GLP-1, ghrelin, etc.) are utilized or not. The application source code can be found on GitHub: https://github.com/geniusserg/Endocrinology (accessed on 1 July 2024). An example of using the developed application for one of the real patients is shown in Figure 6

## 5. Limitations of the Study

The limitations of the study include a small sample size. We have analyzed only a small number of predictors that cannot claim 100% coverage and there is a possibility that a number of more significant predictors are not included in our model. Any model contains only a part of the information that is available to the doctor when examining the patient, so the model can only help in making a decision but not replace the clinical thinking of a specialist.

## 6. Conclusions

In this study, we have successfully identified and ranked indicators for predicting weight loss. Utilizing these predictors, we constructed models with excellent performance metrics. Furthermore, we applied filtering methods to enhance the quality of our models, and our results demonstrated their effectiveness. The models were checked by medical professionals, and valuable feedback was obtained. This indicates the potential for practical application in clinical settings through a user-friendly web application. Additionally, our model can be expanded to accommodate other medications, such as metformin and liraglutide, further increasing its versatility and applicability.

Additionally, despite the less-than-optimal results for a three-month period, our model provides valuable insights. In situations where access to comprehensive analyses is limited, our model enables forecasting of the effects of the medication, albeit with lower accuracy. Notably, our model performs 21% better than random chance, underscoring the effectiveness of the methods applied. Furthermore, it is important to highlight that our model stands as the current best solution for addressing this challenge.

The development of predictive models for weight loss therapy response using artificial intelligence will contribute to increasing the efficiency and effectiveness of medical care delivery. It represents the greatest value for personalized therapy selection, patient time savings, and healthcare cost reduction in both the short and long term. Therefore, the use of these innovative models in personalized and preventive medicine will be in demand in the near future and may eventually become part of routine clinical practice.

## Figures and Tables

**Table 1:** Criteria for inclusion/non-inclusion in the study.

Inclusion Criteria	Non-Inclusion Criteria	Exclusion Criteria
1. Men and women aged 18 years or older; 2. BMI > 30 kg/m^2^; 3. Absence of arterial hypertension (AH) or stable course of hypertension (at the time of inclusion in the study, blood pressure < 140/90 mmHg) without changes in antihypertensive therapy in the past 6 months; 4. Absence of diseases accompanied by thyroid dysfunction; 5. Willingness to follow dietary, physical activity, and therapy recommendations; 6. Documented patient consent to participate in the study.	1. Significant cardiovascular pathology: arterial hypertension (AH) with non-target blood pressure levels, history of myocardial infarction (MI), acute cerebrovascular accident (stroke), angina pectoris, chronic heart failure (CHF) above 2 FC (NYHA), high-risk arrhythmias; 2. Presence of diabetes mellitus; 3. Chronic kidney pathology with glomerular filtration rate (GFR) < 60 mL/min; 4. Liver insufficiency, more than a 3-fold increase in liver transaminases (ALT, AST); 5. Therapy with immunosuppressants, immunomodulators, biological drugs, and other weight-reducing medications received for any reason at the start of the study; 6. Indications of alcohol abuse; 7. History of surgical treatment of obesity.	1. Low patient compliance; 2. The development of adverse reactions during the intervention; 3. Extensive injuries, severe infections, cancer, and other acute conditions.

**Table 2:** Statistical tests results.

Pearson	Mann–Whitney	Chi-Square
Glucagon-like peptide 1 (−0.24, *p*-value—0.04) Ghrelin (ng/mL) (0.25, *p*-value—0.04) miR155 (−0.57, *p*-value—0.04) Procollagen type 1 (−0.37, *p*-value—0.02) NT-proBMP/leptin (−0.41, *p*-value—0.05) sST2/leptin (−0.35, *p*-value—0.05) **for 6 months:** Glomerular filtration rate (0.37, *p*-value—0.04) miR378 (QTT) (−0.51, *p*-value—0.05) MMP-9 (0.56, *p*-value—0.02) Postprandial dynamic of leptin (−0.44, *p*-value—0.01)	Systolic pressure (*p*-value—0.05) Total cholesterol (*p*-value—0.04) Low-density lipoprotein cholesterol (*p*-value—0.04) Glomerular filtration rate (*p*-value—0.05) Glucagon-like peptide 1 (*p*-value—0.01) Ghrelin (*p*-value—0.03) Ghrelin AC calculation by coefficient (*p*-value—0.03) miR378 (QTT) (*p*-value—0.02) miR125 (*p*-value—0.03) miR155 (*p*-value—0.02) **for 6 months:** C-reactive protein (*p*-value—0.05) miR142 (plasma) (*p*-value—0.03) MMP-9 (*p*-value—0.02) Procollagen-1/procollagen-3 (*p*-value—0.05) Postprandial leptin dynamics (*p*-value—0.01)	Postprandial dynamic of leptin (*p*-value—0.02) **for 6 months:** Cardiogroup (*p*-value 0.01)

**Table 3:** Used parameters.

Case	Parameters
3 months	Age, BMI, postprandial leptin dynamic, glucose, glomerular filtration rate, total cholesterol, systolic pressure, C-reactive protein
	*… all from above*, Glucagon-like peptide 1 (GLP-1), glucose-dependent insulinotropic peptide (GIP), ghrelin, miR142 (plasma), sST2
6 months	Weight loss after 3 months, age, BMI, postprandial leptin dynamic, glucose, glomerular filtration rate, total cholesterol, systolic pressure, C-reactive protein
	*… all from above*, Glucagon-like peptide 1 (GLP-1), glucose-dependent insulinotropic peptide (GIP), ghrelin, miR142 (plasma), sST2

**Table 4:** Classification metrics.

Case	Accuracy	F1	Precision	Recall	ROC AUC
3 months	71.14% (±2.91%)	62.31% (±3%)	76.17% (±2.43%)	75% (±3.19%)	0.72 (±0.03)
3 months with scientific parameters	75.34% (±1.84%)	69.92% (±2.39%)	75.44% (±1.45%)	91.49% (±2.24%)	0.75 (±0.02)
6 months	79.55% (±2.95%)	79.53% (±3.01%)	78.26% (±3.19%)	81.82% (±5.77%)	0.87 (±0.04)
6 months with scientific parameters	84.09% (±3.23%)	84.08% (±3.25%)	85.71% (±3.55%)	81.82% (±5.28%)	0.89 (±0.04)

## Data Availability

The data presented in this study are available on request from the corresponding author.
